# Publisher Correction: Rational design of a hospital-specific phage cocktail to treat *Enterobacter cloacae* complex infections

**DOI:** 10.1038/s41564-025-02163-9

**Published:** 2025-10-01

**Authors:** Dinesh Subedi, Fernando Gordillo Altamirano, Rylee Deehan, Avindya Perera, Ruzeen Patwa, Xenia Kostoulias, Denis Korneev, Luke Blakeway, Nenad Macesic, Anton Y. Peleg, Jeremy J. Barr

**Affiliations:** 1 School of Biological Sciences, Monash University, Clayton, Victoria Australia; 2 Centre to Impact AMR, Monash University, Clayton, Victoria Australia; 3 School of Optometry and Vision Science, UNSW Medicine, University of New South Wales NSW, Kensington, New South Wales Australia; 4 Department of Infectious Diseases, The Alfred Hospital and School of Translational Medicine, Monash University, Melbourne, Victoria Australia; 5 Australian Centre for Ecogenomics, School of Chemistry and Molecular Biosciences, University of Queensland, Saint Lucia, Queensland Australia; 6 Infection Program, Monash Biomedicine Discovery Institute, Department of Microbiology, Monash University, Clayton, Victoria Australia; 7 Ramaciotti Centre for Cryo-Electron Microscopy, Monash University, Clayton, Victoria Australia

**Keywords:** Antibiotics, Bacteriophages

Correction to: *Nature Microbiology* 10.1038/s41564-025-02130-4, published online 24 September 2025.

In the version of the article initially published, the “øEnC15” image in Fig. 1e was a duplicate of the “øEnC07” image in Fig. 1d. Fig. 1e has now been corrected to include the correct “øEnC15” image in the HTML and PDF versions of the article.

