# Supplementary material for: Rational design of a hospital-specific phage cocktail to treat Enterobacter cloacae complex infections
Source: Nat Microbiol. 2025 Sep 24;10(11):2702–19. doi: 10.1038/s41564-025-02130-4 (PMC12578640; doi:10.1038/s41564-025-02130-4)
Supplement: Supplementary file 1 — Supplementary Figs. 1–3 and Table 1. [file 41564_2025_2130_MOESM1_ESM.pdf]

nature microbiology

Supplementary information https://doi.org/10.1038/s41564-025-02130-4

Rational design of a hospital-specific phage cocktail to treat *Enterobacter cloacae* complex infections

In the format provided by the authors and unedited

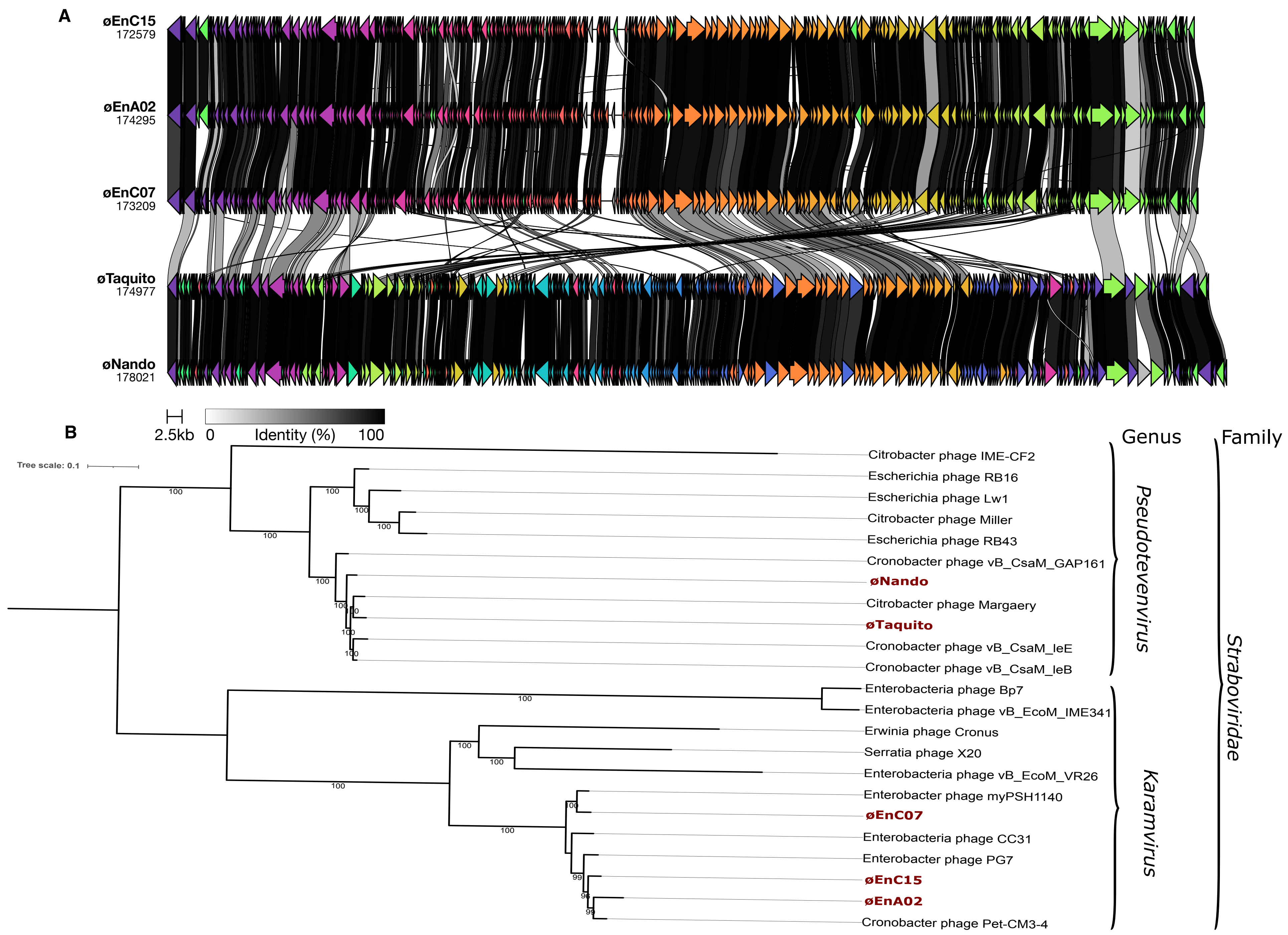

Supplementary Figure 1. Comparative genome analysis and phylogenetic relationships of phages in the phage cocktail. (A) Whole-genome BLAST comparison of the five phages included in the cocktail, visualized using Clinker (https://github.com/gamcil/clinker).Genome sizes (in base pairs) are indicated. (B) Maximum likelihood phylogenetic tree of phages belonging to the Karamvirus and Pseudotevenvirus genera within the Straboviridae family, based on whole-genome alignment. Branch support values are shown as bootstrap percentages. Cocktail phages are highlighted in red.

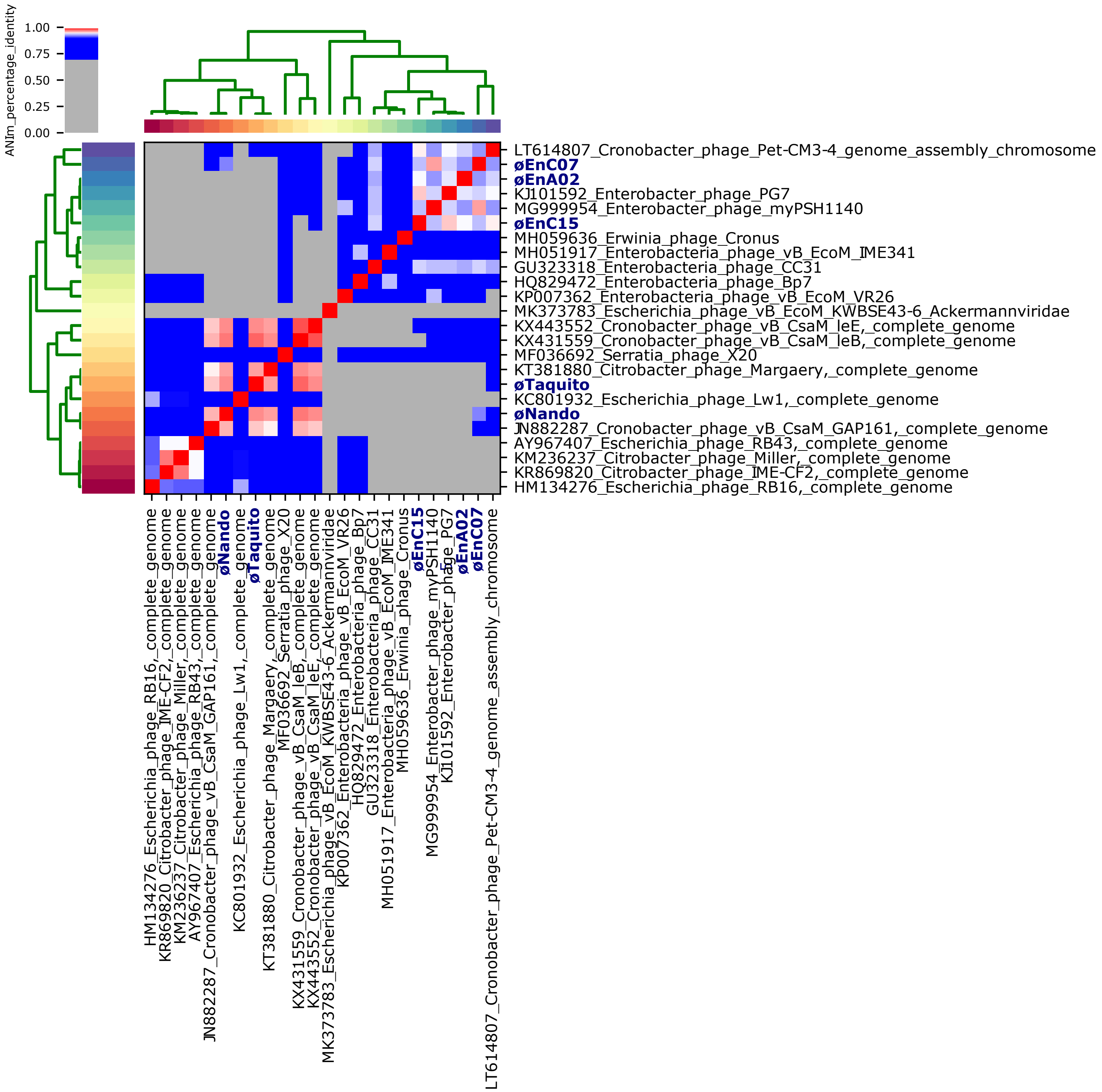

Supplementary Figure 2. Average nucleotide identity of cocktail phages with their close match of the genus Karamvirus and Pseudotevenvirus genera within Straboviridae.

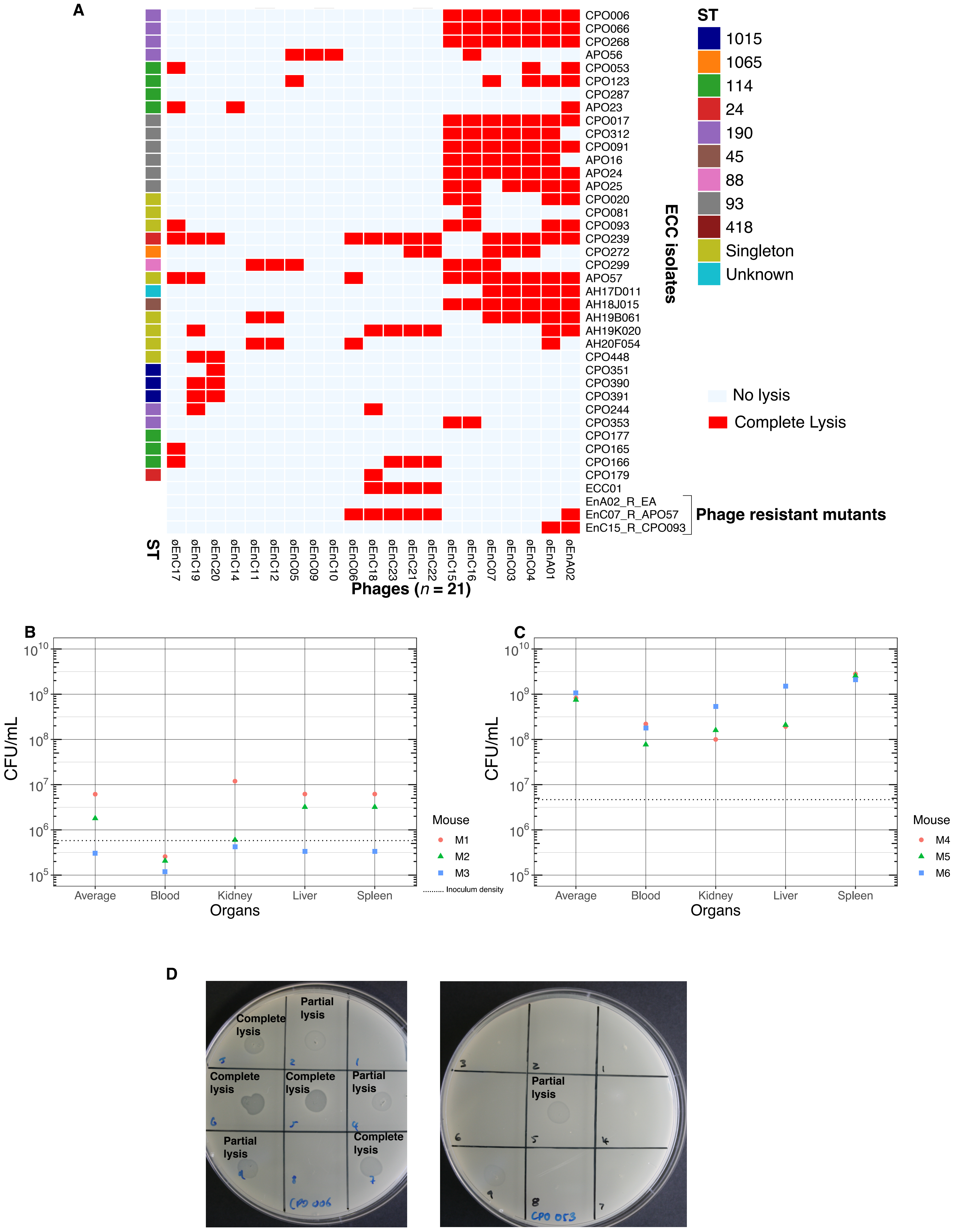

Supplementary Figure 3. (A) Host range map of the phages against wild-type (n = 36) and phage-resistant mutants (n = 3). The rows show the bacterial isolates, and the columns show the isolated phages. Red means that the phage completely lysed the host and light blue means no lysis of the host. (B) Colonisation of ECC isolate APO57 in different mouse organs 12 hours post intraperitoneal infection. Mice were injected with 8.1 × 10^5 CFU of APO57 per mouse (dotted line). Bacterial loads were measured in various organs 12 hours post-infection. Mouse 3 cleared the infection, while low-level colonization was observed in mice 1 and 2. (C) Colonisation of ECC isolate APO57 in mouse organs 12 hours post intraperitoneal infection.Mice were injected intraperitoneally with 4.67 × 10^6 CFU of ECC per mouse (dotted line). At 24 hours post-infection (hpi), all mice showed clinical signs and consistent bacterial colonization across vital organs. Dots represent data form each mouse (n = 3). (D) Representative photograph of spot plate assay on ECC isolates and classification of complete and partial lysis due to phage infection.

Supplementary Table 1: Plasmids and primers used in the complementation experiments

**Plasmid or Host**	**Forward Primer (5ʹ-3ʹ)**	**Reverse Primer (5ʹ-3ʹ)**	**Restriction sites**
pBBR1MCS-2 (Kan)	TTCCATTCGCCATTCAGGCT	GGAATTGTGAGCGGATAACA	–
APO57	AGACACACTAGTATGCTGTCGAAGTGCTGCATC	AGTAATGTCGACGTGATGATGTCAAGATGACCG	SpeI/SalI
CPO093	CGAATCGAATTCCGTCCTTGACAATCCTCCGC	ACTAGCACTAGTGGCAGTGCTACTATCGCTGC	EcoR1/SpeI
Eaeru (OmpW)	AAGCGCGGATCCCAGAACGTTACTGAGTGCTGC	ACACATGTCGACCACGCCCATCGGTTGGTCTG	BamHI/ SalI
Eaeru(wba)	AAGCGCGGATCCTGCAGAAACAACGCCTTGCG	AAGCGCGGATCCTGCAGAAACAACGCCTTGCG	BamHI/HindIII
