# Supplementary material for: Rational design of a hospital-specific phage cocktail to treat Enterobacter cloacae complex infections
Source: Nat Microbiol. 2025 Sep 24;10(11):2702–19. doi: 10.1038/s41564-025-02130-4 (PMC12578640; doi:10.1038/s41564-025-02130-4)
Supplement: Supplementary file 2 — Reporting Summary [file 41564_2025_2130_MOESM2_ESM.pdf]

nature portfolio

Corresponding author(s): Jeremy Barr

Last updated by author(s): Aug 7, 2025

Reporting Summary

Nature Portfolio wishes to improve the reproducibility of the work that we publish. This form provides structure for consistency and transparency in reporting. For further information on Nature Portfolio policies, see our Editorial Policies and the Editorial Policy Checklist.

Statistics

For all statistical analyses, confirm that the following items are present in the figure legend, table legend, main text, or Methods section.

n/a	Confirmed	
☐	☒	The exact sample size (*n*) for each experimental group/condition, given as a discrete number and unit of measurement
☐	☒	A statement on whether measurements were taken from distinct samples or whether the same sample was measured repeatedly
☐	☒	The statistical test(s) used AND whether they are one- or two-sided<br>*Only common tests should be described solely by name; describe more complex techniques in the Methods section.*
☐	☒	A description of all covariates tested
☐	☒	A description of any assumptions or corrections, such as tests of normality and adjustment for multiple comparisons
☐	☒	A full description of the statistical parameters including central tendency (e.g. means) or other basic estimates (e.g. regression coefficient) AND variation (e.g. standard deviation) or associated estimates of uncertainty (e.g. confidence intervals)
☐	☒	For null hypothesis testing, the test statistic (e.g. *F*, *t*, *r*) with confidence intervals, effect sizes, degrees of freedom and *P* value noted<br>*Give P values as exact values whenever suitable.*
☒	☐	For Bayesian analysis, information on the choice of priors and Markov chain Monte Carlo settings
☒	☐	For hierarchical and complex designs, identification of the appropriate level for tests and full reporting of outcomes
☒	☐	Estimates of effect sizes (e.g. Cohen's *d*, Pearson's *r*), indicating how they were calculated

Our web collection on statistics for biologists contains articles on many of the points above.

Software and code

Policy information about availability of computer code

Data collection	The data for this study was collected using lab experiments. For the comparison and analysis of the bacteriophage genome, we utilised the NCBI genome database and BLAST search. Information on bacteriophages classification was gathered from the International Committee on Taxonomy of Viruses. No special software was used to collect the data.
Data analysis	GraphPad Prism 10 and R 4.2.3 with RStudio 2024.04.1 using recent versions of publicly available packages.

For manuscripts utilizing custom algorithms or software that are central to the research but not yet described in published literature, software must be made available to editors and reviewers. We strongly encourage code deposition in a community repository (e.g. GitHub). See the Nature Portfolio guidelines for submitting code & software for further information.

Data

Policy information about availability of data

All manuscripts must include a data availability statement. This statement should provide the following information, where applicable:

- Accession codes, unique identifiers, or web links for publicly available datasets
- A description of any restrictions on data availability
- For clinical datasets or third party data, please ensure that the statement adheres to our policy

All raw data, supplementary materials, and analysis data generated during this study are publicly available at https://github.com/ECCphage. Additional supporting

data, figures, and tables are provided within the Article and its Extended Data and Supplementary Information. Genome sequences have been deposited in the NCBI database under BioProject accession number PRJNA629076. All data are freely accessible for research purposes.

Research involving human participants, their data, or biological material

Policy information about studies with human participants or human data. See also policy information about sex, gender (identity/presentation), and sexual orientation and race, ethnicity and racism.

Reporting on sex and gender	This information has not been collected. Not applicable for this study.
Reporting on race, ethnicity, or other socially relevant groupings	This information has not been collected. Not applicable for this study.
Population characteristics	This information has not been collected. Not applicable for this study.
Recruitment	This information has not been collected. Not applicable for this study.
Ethics oversight	This information has not been collected. Not applicable for this study.

Note that full information on the approval of the study protocol must also be provided in the manuscript.

Field-specific reporting

Please select the one below that is the best fit for your research. If you are not sure, read the appropriate sections before making your selection.

☒ Life sciences ☐ Behavioural & social sciences ☐ Ecological, evolutionary & environmental sciences

For a reference copy of the document with all sections, see nature.com/documents/nr-reporting-summary-flat.pdf

Life sciences study design

All studies must disclose on these points even when the disclosure is negative.

Sample size	No proper sample size was calculated for the bacterial isolates. We used the entire population of isolates available at the study site (The Alfred Hospital in Melbourne, Australia). A subset of these isolates, selected based on their genetic diversity, was used in various experiments as detailed in the manuscript. For the animal study, a sample size of at least six animals per group (treatment and control) was calculated to achieve a power of 90% with a significance level of 0.05.
Data exclusions	No data were excluded from the study.
Replication	The experiments were performed in replicates on at least three different occasions, all attempts at replication were successful, and all data are presented with error bars or whiskers in box plots where applicable.
Randomization	Animals were randomly allocated into control and treatment groups.
Blinding	Blinding was not relevant to the animal study as bacterial and phage counts were objectively measured after each experiment. Therefore outcomes were not influenced by human judgment. For qualitative assessments of the spot assay by phages, evaluations were conducted by at least two blinded researchers.

Reporting for specific materials, systems and methods

We require information from authors about some types of materials, experimental systems and methods used in many studies. Here, indicate whether each material, system or method listed is relevant to your study. If you are not sure if a list item applies to your research, read the appropriate section before selecting a response.

Materials & experimental systems

n/a	Involved in the study	
☒	☐	Antibodies
☒	☐	Eukaryotic cell lines
☒	☐	Palaeontology and archaeology
☐	☒	Animals and other organisms
☒	☐	Clinical data
☒	☐	Dual use research of concern
☒	☐	Plants

Methods

n/a	Involved in the study	
☒	☐	ChIP-seq
☒	☐	Flow cytometry
☒	☐	MRI-based neuroimaging

Animals and other research organisms

Policy information about studies involving animals; ARRIVE guidelines recommended for reporting animal research, and Sex and Gender in Research

Laboratory animals	BALB/c mice
Wild animals	The study did not involve wild animal.
Reporting on sex	The choice to use female mice in septicemia models was based on our previous study, which showed that they provide consistent data, are easier to handle, and exhibit reduced aggression.
Field-collected samples	This Study didn't involve field collected samples.
Ethics oversight	All protocols involving animals were reviewed and approved by the Monash University Animal Ethics Committee (Project ID: 2022-27681-80733). The animals were housed at the Monash Animal Research Facility, Monash University.

Note that full information on the approval of the study protocol must also be provided in the manuscript.

Plants

Seed stocks	Not applicable
Novel plant genotypes	Not applicable
Authentication	Not applicable
