# Supplementary figures and images for: Rational design of a hospital-specific phage cocktail to treat Enterobacter cloacae complex infections
Source: Nat Microbiol. 2025 Sep 24;10(11):2702–19. doi: 10.1038/s41564-025-02130-4 (PMC12578640; doi:10.1038/s41564-025-02130-4)

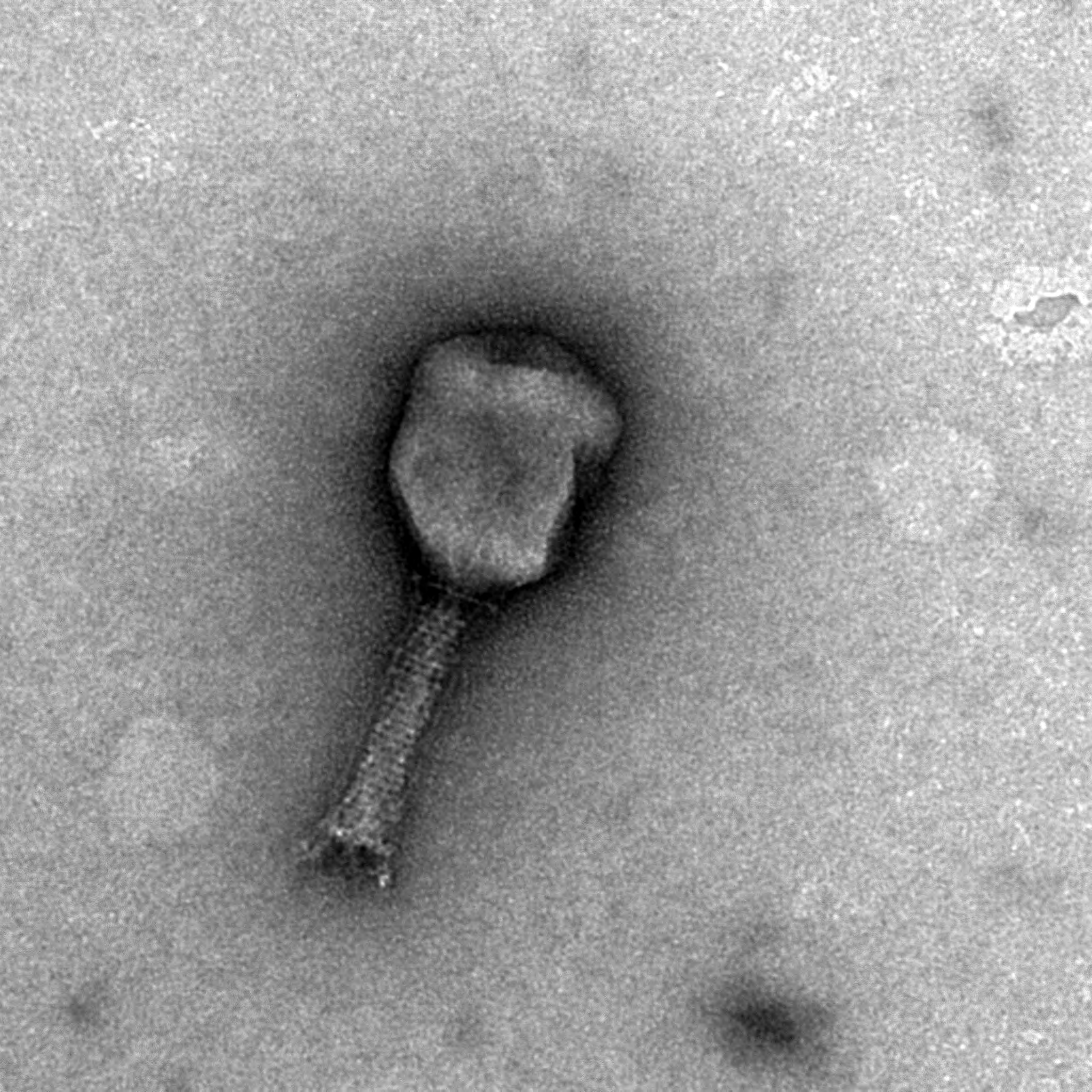

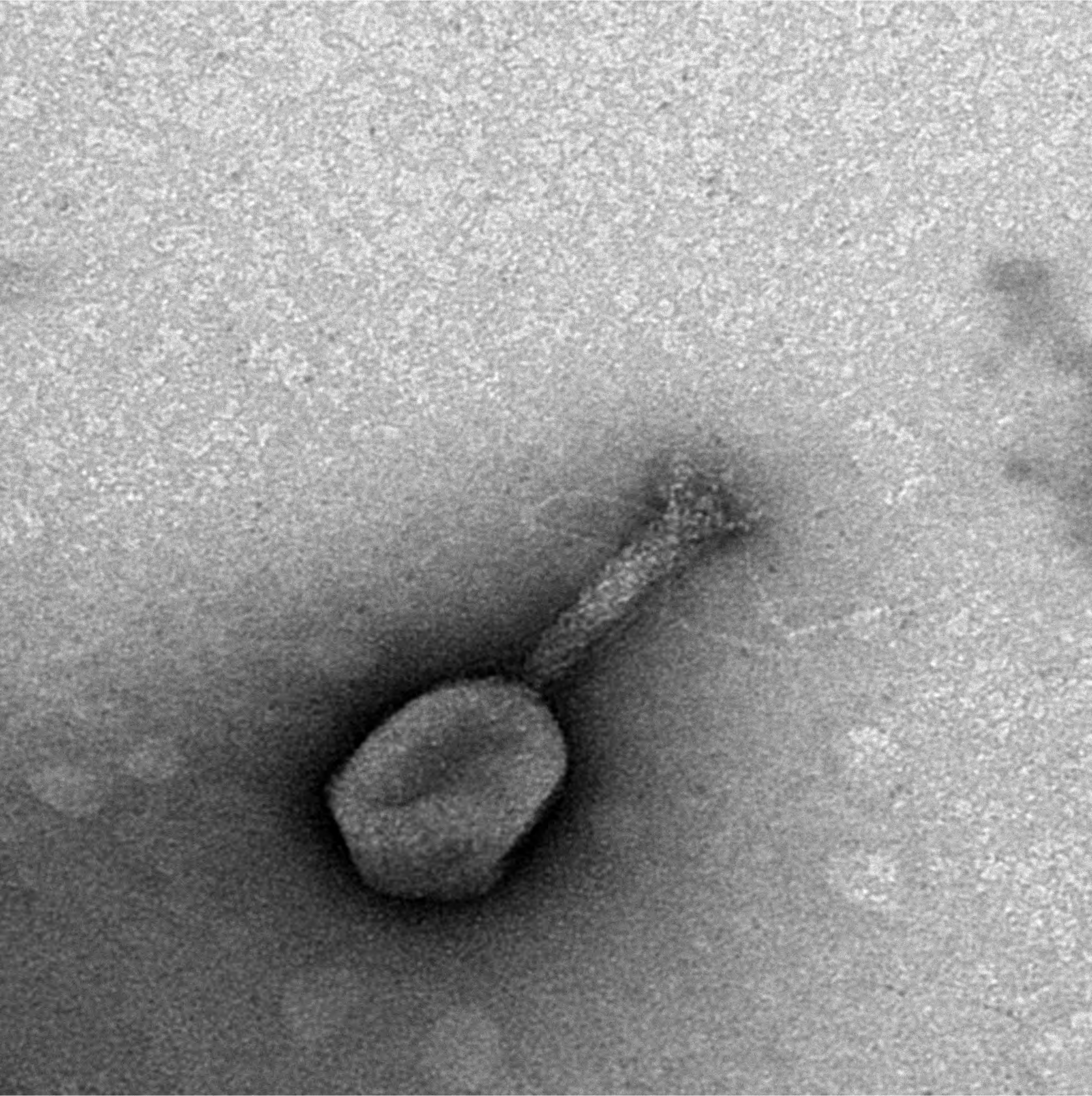

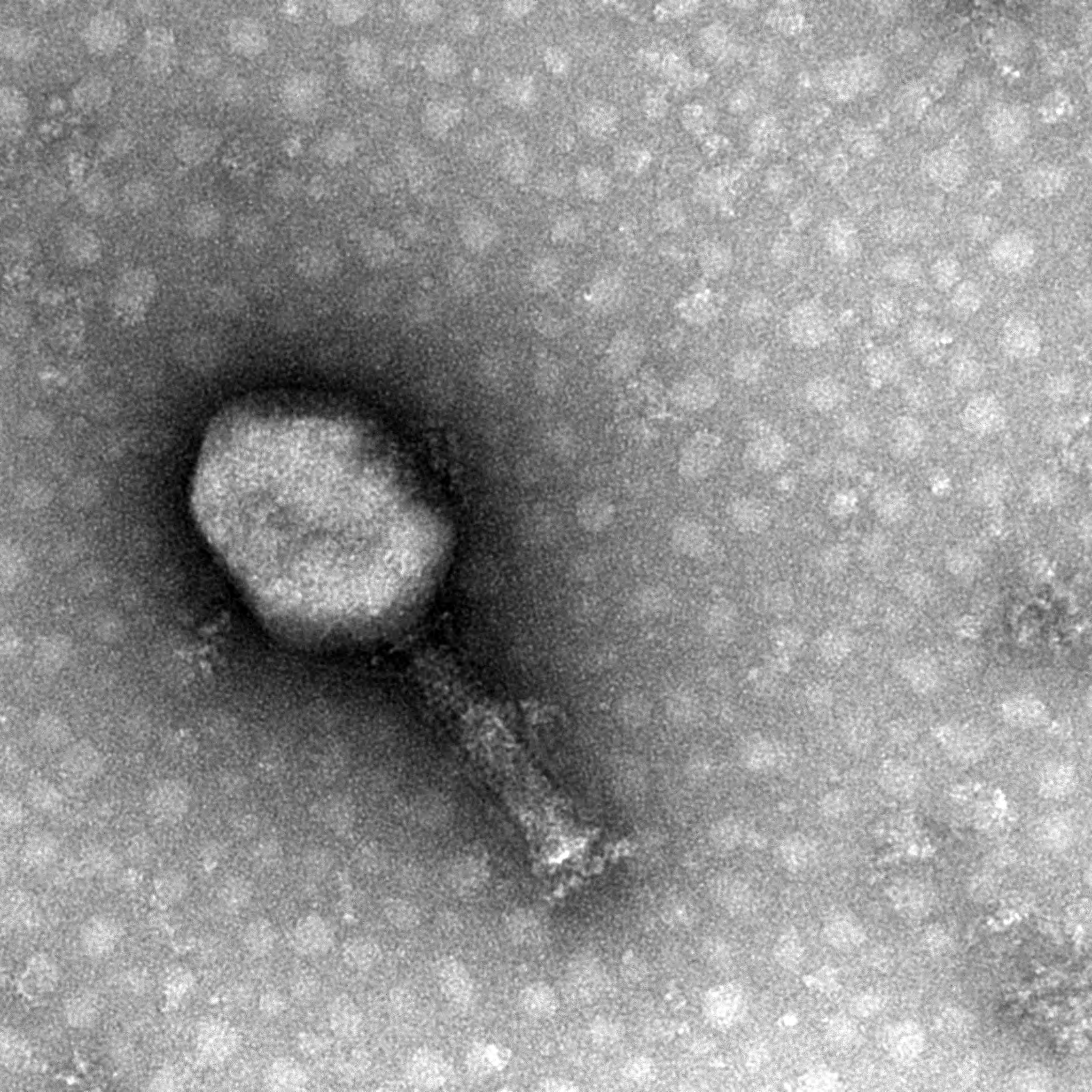

Supplement: Supplementary file 12 — TEM images associated with Fig. 1. [file 41564_2025_2130_MOESM12_ESM.pdf]

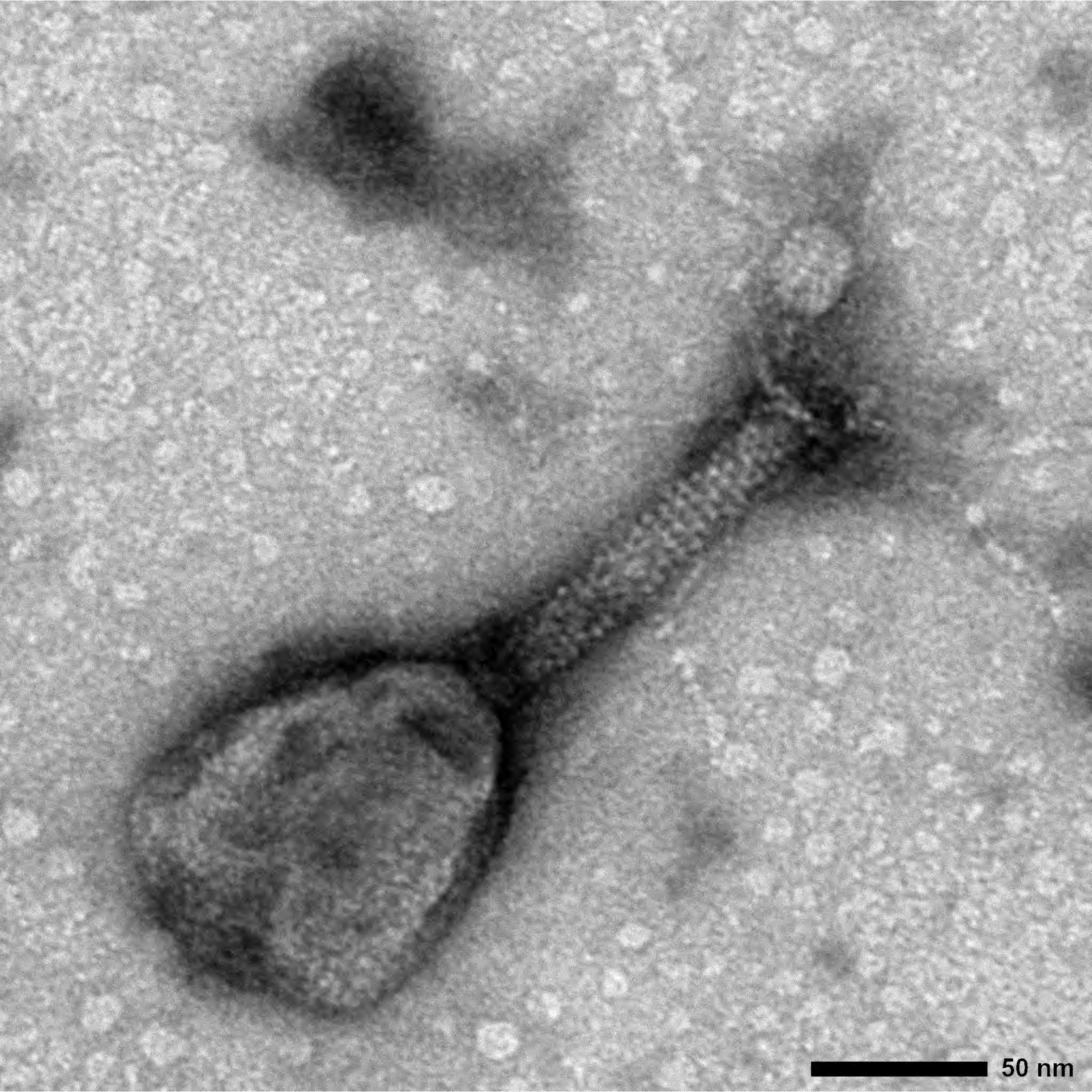

50 nm

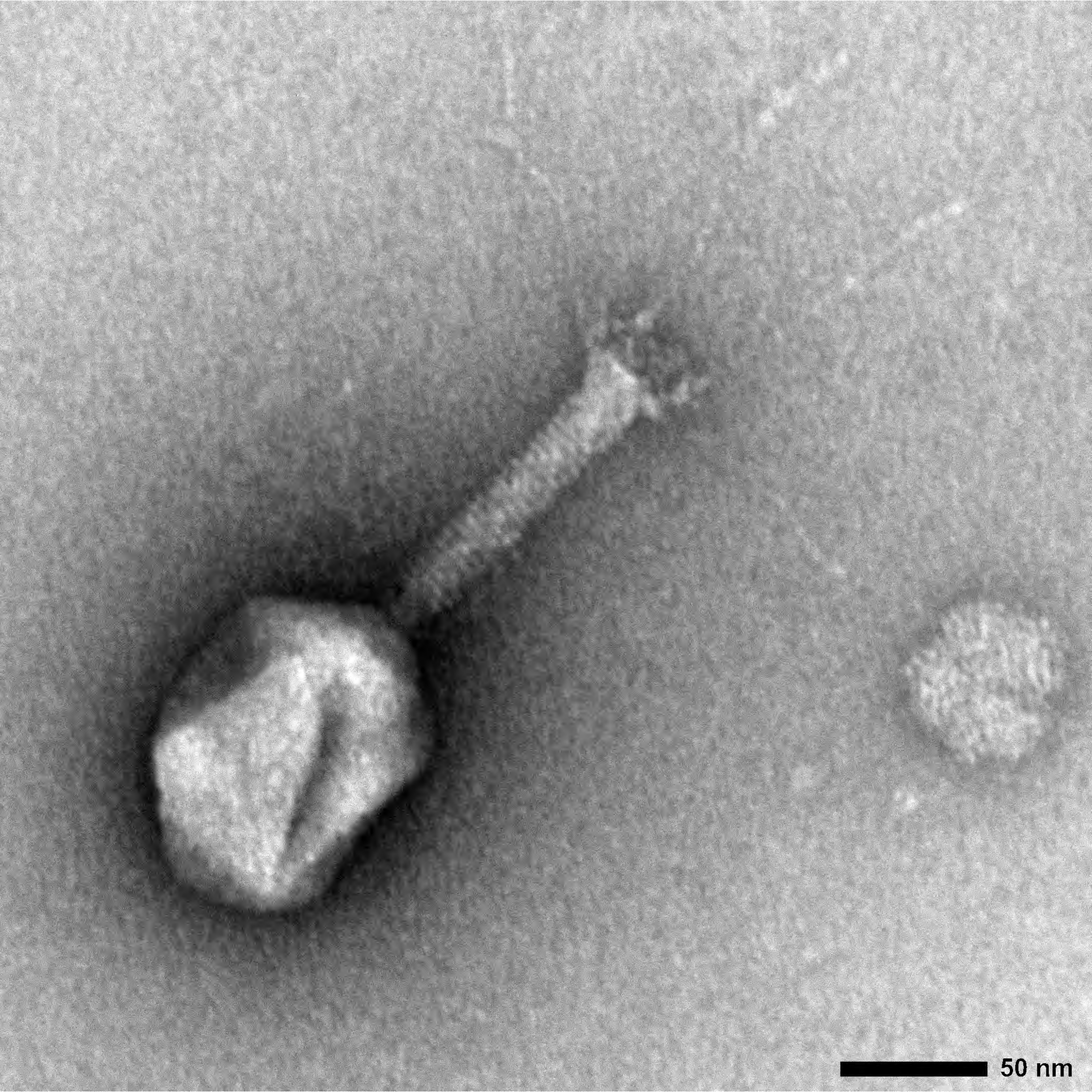

50 nm

Supplement: Supplementary file 13 — TEM images associated with Fig. 4. [file 41564_2025_2130_MOESM13_ESM.pdf]

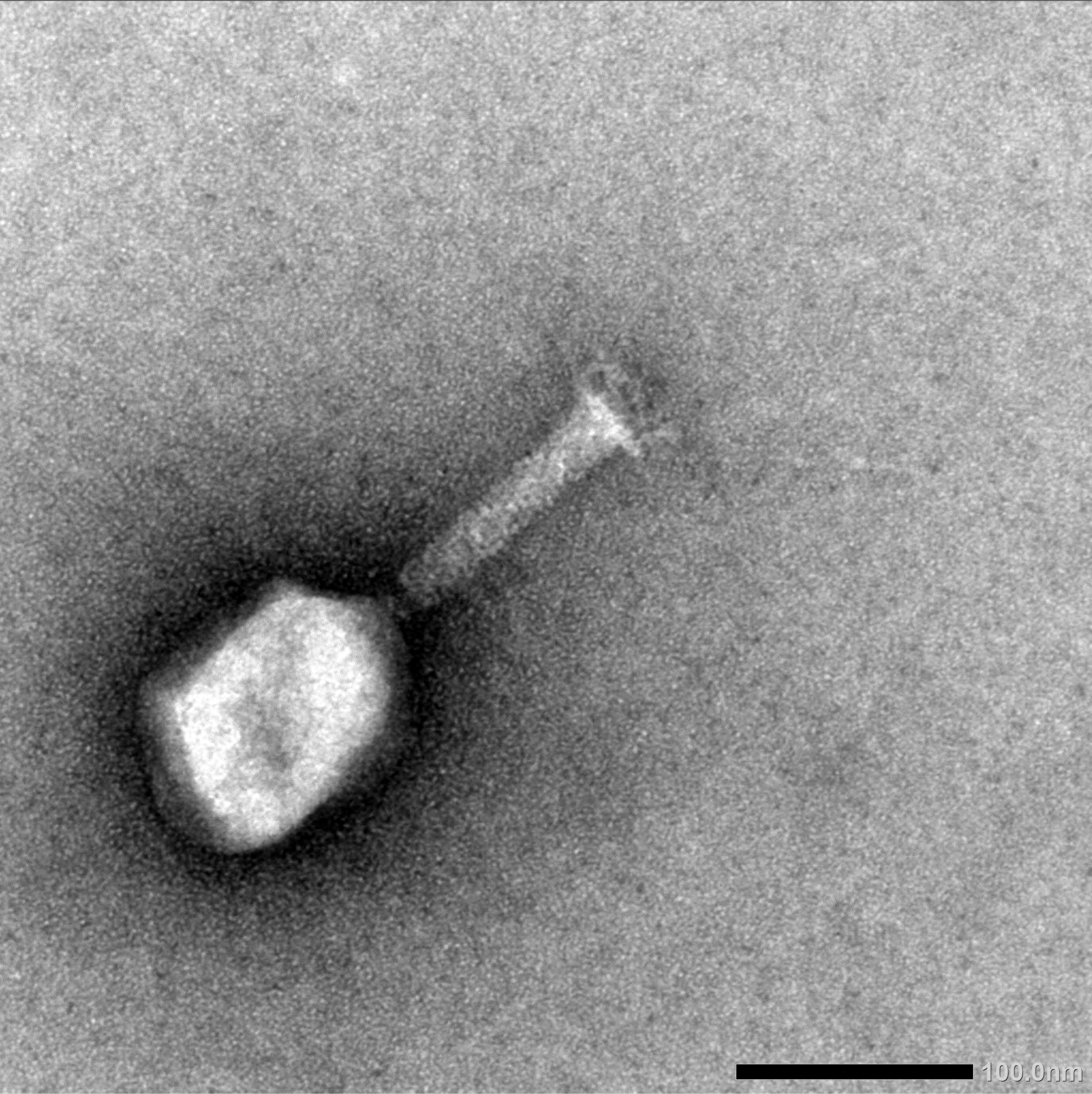

100.0nm

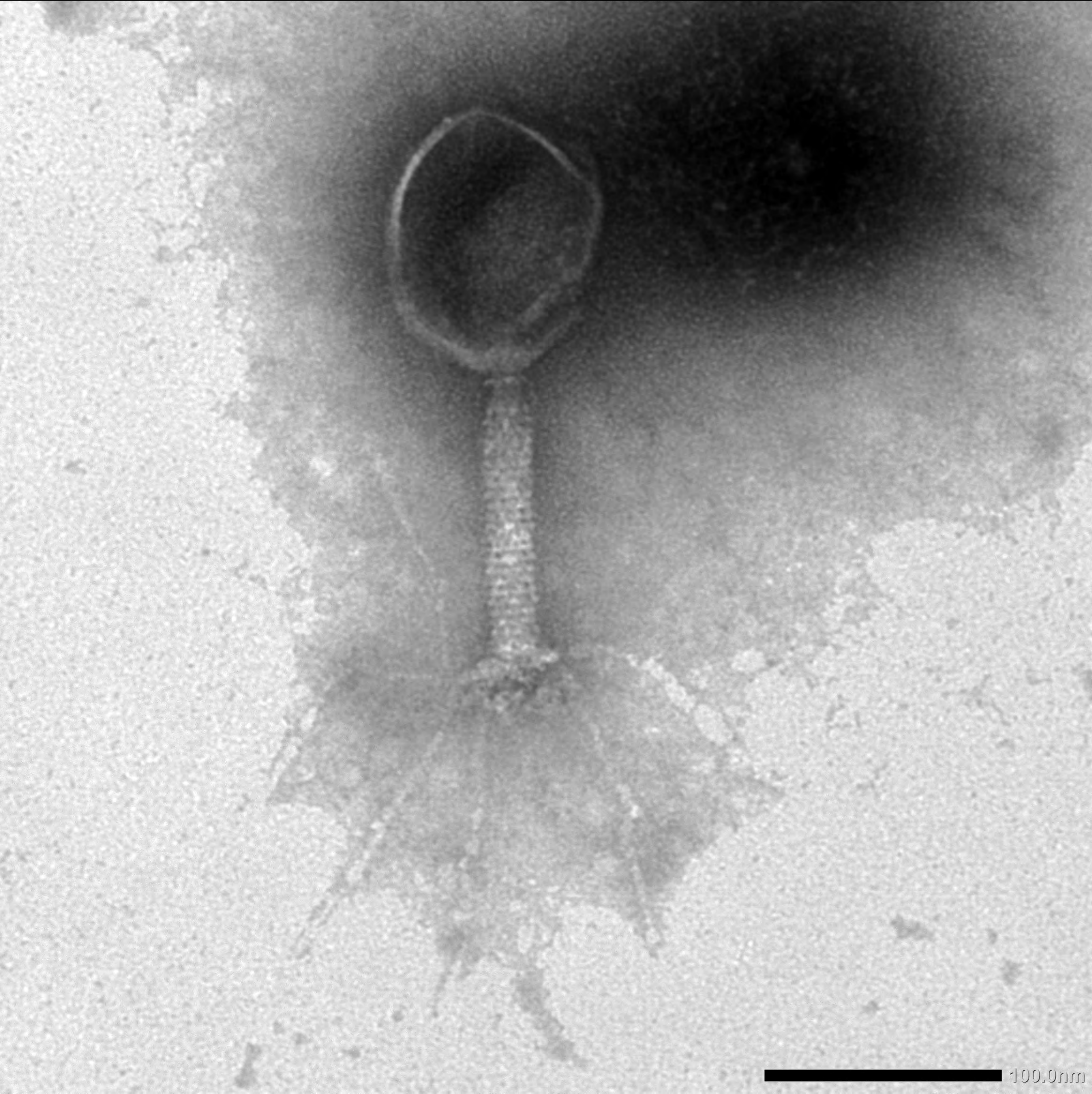

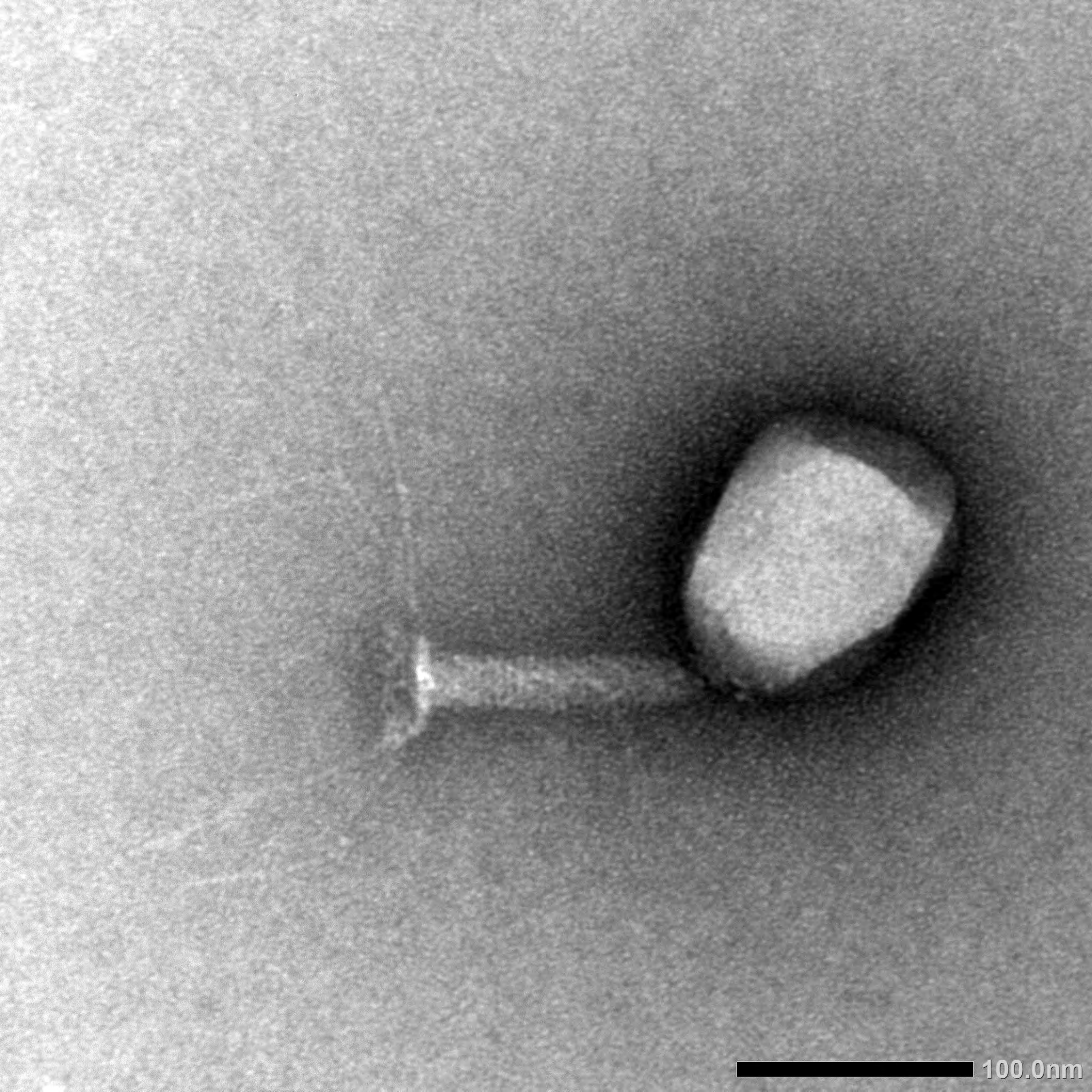

100.0nm

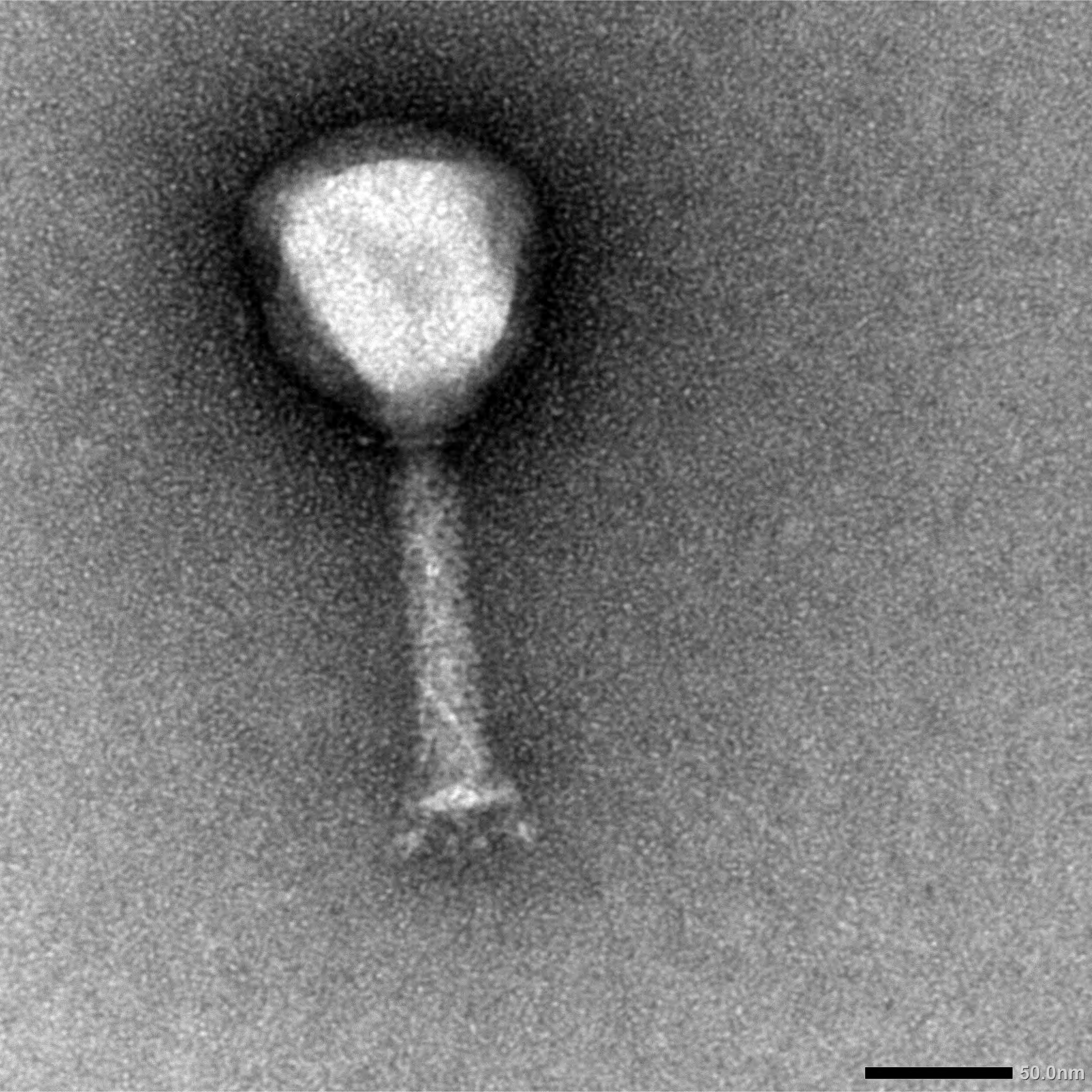

50.0nm

Supplement: Supplementary file 14 — TEM images associated with Fig. 5. [file 41564_2025_2130_MOESM14_ESM.pdf]
